# NEIGHBORHOOD STREET CONNECTIVITY, FOOD ACCESS, AND MORTALITY AMONG OLDER ADULTS: EDUCATION AND GENDER DIFFERENCES

**DOI:** 10.1093/geroni/igae098.2664

**Published:** 2024-12-31

**Authors:** Pui Yin Cheung, David Curtis, Ming Wen

**Affiliations:** University of Hong Kong, Pok Fu Lam, Hong Kong; University of Utah, Salt Lake City, Utah, United States; University of Hong Kong, Pok Fu Lam, Hong Kong

## Abstract

Disadvantages in multiple dimensions have garnered increasing attention with strong empirical support. This study examines whether the intersection between education and gender reveals novel neighborhood effects. Two particular areas of neighborhood built and service environments are examined: pedestrian-oriented street connectivity and residing in a low-food-access tract. They are important for health among older adults, given their limited functioning and health challenges. We examined the second and third waves of the MIDUS study, restricted to those aged 65 or above in the second wave. We estimated a series of discrete-time hazard models with 12,141 person-years across 15 years and included individual-level sociodemographic control variables. We also controlled for tract-level characteristics: urban/rural categories, median household income, percentages with college or above education, and racial/ethnic percentages. Interaction between any single dimension of disadvantage and pedestrian-oriented street connectivity didn’t reveal significant differential relationships with mortality. However, better street connectivity was associated with less hazard of mortality among less-educated men once three-way interactions between education, gender, and street connectivity were introduced. In contrast, well-educated older adults residing in a low-food-access tract had a significantly higher hazard of mortality than those not residing in such a tract. The model with three-way interactions revealed more adverse effects of residing in a low-food-access tract among well-educated men only. The findings reveal mortality differences by education and health and their interactions with neighborhood characteristics despite no apparent differences based on a single dimension of disadvantage, supporting the usefulness of the perspective of disadvantages in multiple dimensions.

